# The Evolving Paradigm in the Management of Intracranial Atherosclerotic Disease

**DOI:** 10.1155/2012/289852

**Published:** 2011-12-19

**Authors:** Ali K. Ozturk, Ketan R. Bulsara

**Affiliations:** Department of Neurosurgery, Yale School of Medicine, New Haven, CT 06510, USA

## Abstract

Intracranial atherosclerotic disease (ICAD) is a major cause of ischemic stroke worldwide and represents a significant health problem. The pathogenesis and natural history of ICAD are poorly understood, and rigorous treatment paradigms do not exist as they do for extracranial atherosclerosis. Currently, the best treatment for ICAD remains aspirin therapy, but many patients who are placed on aspirin continue to experience recurrent strokes. As microsurgical and endovascular techniques continue to evolve, the role of extracranial to intracranial bypass operations and stenting are increasingly being reconsidered. We performed a PubMed review of the English literature with a particular focus on treatment options for ICAD and present evidence-based data for the role of surgery and stenting in ICAD against medical therapy alone.

## 1. Introduction

Intracranial atherosclerotic disease (ICAD) is the process by which atherosclerotic plaques affect large intracranial arteries. Intracranial stenosis represents the most advanced stage of ICAD and is a precursor to ischemic stroke. ICAD is the leading cause of stroke among patients of Asian ancestry [1], and Hispanics and Africans also appear to be more prone to [2] intracranial as opposed to extracranial atherosclerosis. Whites, on the other hand, are less affected, but ICAD is still thought to account for almost 10% of ischemic strokes in this subpopulation [3]. Thus, worldwide, ICAD may be the leading the cause of ischemic stroke.

 Atherosclerotic lesions, as elsewhere in the body, develop silently and insidiously over years prior to becoming suddenly symptomatic in the form of a stroke. Symptomatic ICAD is burdened with an unacceptably high recurrence rate, such that among patients with symptomatic ICAD and >70% stenosis, approximately 23% will have a recurrent stroke over the ensuing 12 months [4], and nearly half of these recurrent strokes tend to be disabling. The prevalence and natural history of asymptomatic ICAD are much less understood, particularly in people of European descent.

 Due to this lack of insight, rigorous treatment paradigms do not exist for ICAD as they do for extracranial atherosclerotic disease. The treatment strategies for ICAD include optimal medical management, surgical, and endovascular options. In this paper, we aim to define the optimal treatment strategies for this devastating disease.

## 2. Methods

MEDLINE and PubMed searches of the English literature were performed with the following keywords: intracranial atherosclerosis, extracranial-intracranial bypass, intracranial stenting, Wingspan, drug-eluding stent, stroke, and medical therapy. The relevant literature was reviewed and was supplemented as necessary from the bibliography of selected articles, with a particular focus on articles which discussed therapeutic interventions for ICAD.

## 3. Results

### 3.1. Optimal Medical Management

The results of the Warfarin-Aspirin Symptomatic Intracranial Disease (WASID) study not only demonstrated the role for medical therapy in ICAD, but also provided important information regarding the natural history. Patients presenting within 90 days of a transient ischemic attack (TIA) or nonsevere stroke attributable to angiographically proven high-grade (50–99%) stenosis of a major intracranial artery were given either aspirin or warfarin and were followed for the primary endpoints of ischemic stroke, hemorrhagic stroke, and vascular death. Based on this data, warfarin did no better than aspirin in stroke prevention but was associated with significantly higher rates of adverse events, such that 8.3% of patients randomized to warfarin had one episode of major hemorrhage compared to 3.2% of patients randomized to aspirin therapy [4].

 Based on the WASID data, aspirin is the antithrombotic drug of choice in ICAD. More recent evidence suggests that dual antiplatelet therapy may be more effective than aspirin alone in preventing microembolic signals detected with transcranial Doppler ultrasound with similar adverse events in both arms [5]. Further evidence is needed to determine if dual antiplatelet therapy is indeed superior to aspirin alone in preventing clinical strokes in patients with symptomatic ICAD.

 Although the WASID trial was not designed to study the importance of risk factor control, several important conclusions are reached from its substudies. Thus, while lowering blood pressure during followup appears to reduce recurrence risk [6], the effects of lipid management seem more controversial [7]. Further studies will be needed to clarify the role of risk factor management in these patients.

### 3.2. EC-IC Bypass

**Figure 1:**
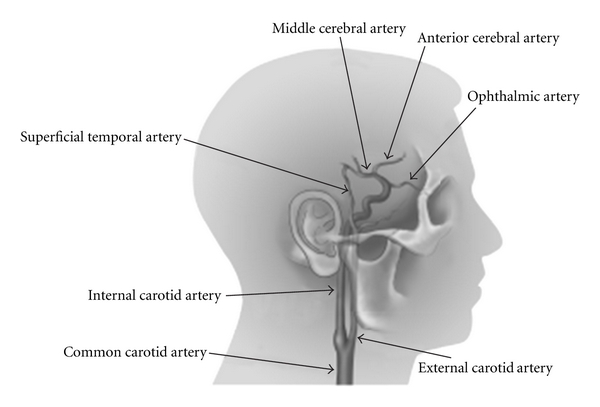
Schematic of STA-MCA bypass.

Flow augmentation in the setting of anterior circulation ischemia can be achieved surgically via external carotid to internal carotid (EC-IC) bypass procedures. Typically, the superficial temporal artery (STA) is anastomosed to the middle cerebral artery (MCA) provided sufficient flow is obtained via the STA (Figure 1). If the STA demonstrates insufficient flow, the cervical carotid can be used via an interposition graft, such as the saphenous vein.

The EC-IC study published in 1985 [8] quickly led to a sharp decline in the use of this intervention for anterior circulation ICAD, since it failed to demonstrate any reduction in strokes compared to best medical management. Briefly, 1377 patients were randomized to surgery plus medical management versus medical management alone. Despite bypass patency rates of 96% and a relatively low complication rate of 3%, long-term followup at 55 months revealed no benefit with regards to stroke prevention [8].

 Upon closer examination, several shortcomings become apparent in this study. First and foremost, patients who had ICAD who were not amenable to carotid endarterectomy and who demonstrated symptoms were included in the study, with disregard toward hemodynamic compromise. Thus, patients who may have had disease due to embolic phenomenon and small vessel disease who would not have benefited from EC-IC were indiscriminately included in the study. Patients with stage 2 hemodynamic insufficiency (i.e., those with a higher oxygen extraction fraction (OEF)), have been shown to have a 26% versus 5% risk of stroke at 2 years compared to those with normal OEF as detected by PET measurement [9, 10]. Second, many patients who underwent surgery did so outside of the trial, potentially implicating that those who needed the surgery more urgently were omitted, thus diluting the beneficial effects of the procedure.

 In lieu of these criticisms, the Carotid Occlusion Surgery Study (COSS) [11] and the Japanese EC-IC Bypass Trial (JET) were underway in the United Stated and Japan, respectively, to assess the potential for a newfound usefulness of this operation if the patients are selected based on a more sophisticated analysis of hemodynamic compromise. At the time of writing this paper, the results of these trials have not been published; however, COSS was terminated prematurely due to lack of benefit in patients undergoing EC-IC bypass.

 The excimer laser-assisted nonocclusive anastomosis (ELANA) is a novel bypass technique which critically omits the use of temporary occlusion using a suction laser catheter which produces an arteriotomy only after the anastomosis is created [12]. While the main use of this technique has been in surgery for complex intracranial aneurysms, recent work is starting to allude to a potential role for it in ICAD. In a recent publication, van Doormaal et al. used ELANA in performing high-flow EC-IC bypasses in 24 patients with TIA or minor strokes in the setting of carotid artery occlusion. All patients had a successful bypass procedure. Two patients suffered a fatal intracerebral hemorrhage within 30 days of the procedure. During an average followup of 4 years, 18 of the surviving 22 patients had patent bypasses and were free of recurrent symptoms. In the remaining 4, the bypass had occluded causing ischemic symptoms [13]. Larger, more specific studies are needed to investigate the potential use of this novel technique in the treatment of ICAD.

### 3.3. Endovascular Therapy

Endovascular devices and expertise using them continue to improve at a rapid pace, and they represent a promising new approach to treating symptomatic ICAD. Initially, endovascular therapy was limited to percutaneous transluminal angioplasty (PTA), but this fell out of favor due to periprocedural morbidity and mortality along with significant and highly problematic rates of restenosis. The chief cause of morbidity using PTA alone comes from vessel dissection, ranging from 14–50% reported in the literature [14, 15]. In addition, over one quarter (27%) of treated vessels will demonstrate restenosis at 5 months following treatment [14, 16]. Ultimately, angioplasty proved to be only a temporary fix.

 The advent of stents, which have the capacity to not only increase tissue perfusion but also remodel the diseased vasculature, is an area of increasing promise. Procedural success rates, periprocedural morbidity and mortality, and restenosis rates are the chief parameters that are used to compare different stent types in the use of ICAD and are the subject of much research and controversy.

Drug-eluting stents (DESs) were shown to greatly decrease the restenosis rates in coronary artery disease [17], and there have been attempts to duplicate this success in the treatment of ICAD. Initial work using DES in intracranial disease by Abou-Chebl et al. [18] treating a small number of patients demonstrated a similarly promising decrease in short-term restenosis rates compared to bare metal stents. However, due to the known risk of delayed stent thrombosis, combined with the lack of long-term data, DES in the treatment of ICAD has largely fallen out of practice.

The balloon expandable Pharos stent has showed initial promise in two small studies in the treatment of ICAD. In a retrospective study, Freitas et al. treated 32 patients with >50% stenosis using the Pharos stent with a 96% success rate. The 30-day clinical followup revealed 2 patients who had suffered ipsilateral stroke and 3 patients who had died, though 2 of the deaths were likely due to medication noncompliance. Ten-month clinical followup, however, revealed no additional strokes or deaths, and only four clinically silent restenosis [19]. A second study from Germany enrolled 21 patients with >70% stenosis, 7 of which were treated in the setting of acute stroke. They achieved a 90% clinical success rate. Of the electively treated patients (14), 3 went on to suffer a stroke or die during an average clinical followup of 7 months. Interestingly, of the 7 patients treated acutely, 2 died within the first 30 days, and another 2 went on to develop ipsilateral strokes over the following 10 months [20], reiterating the risk of intracranial stenting in the setting of acute stroke.

**Figure 2:**
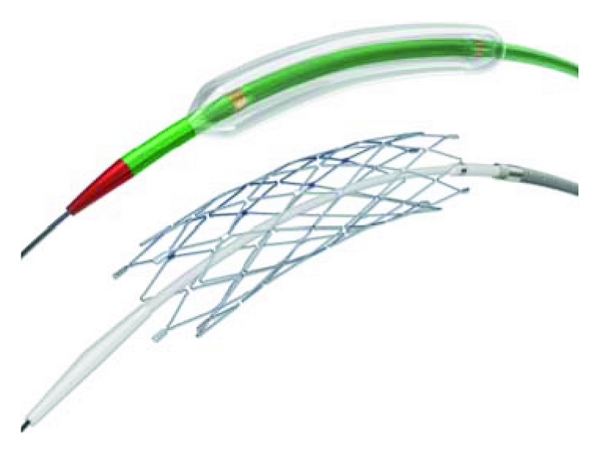
Stent and balloon catheter commonly used in ICAD.

The flexible Wingspan stent was approved for use in intracranial vessels with >50% stenosis in 2005. The flexibility afforded by this stent increases its usefulness in the tortuous intracranial vasculature (Figure 2), and technical success rates using the Wingspan stent have been reported to be as high as 97-98% [21]. Guo et al. reported a 98% technical success rate specifically for MCA stenosis [22], a vessel traditionally challenging to treat endovascularly. A prospective Wingspan study, however, demonstrated an approximately 30% rate of in-stent stenosis [23], higher than what had previously been reported in the neurosurgical literature. While most of these were clinically silent, 5% led to significant morbidity and mortality [23]. In a recent review, Ding and Liu [24] found the Wingspan stent to have an overall higher technical success rate in the literature and possibly a lower complication rate, although the authors admit that shorter clinical followup for the Wingspan studies made the comparison uneven.

 Due to the initially promising and conflicting results of the Wingspan stent, the SAMMPRIS trial was designed to compare the Wingspan stent and medical therapy to aggressive medical therapy alone in symptomatic ICAD patients randomizing patients in 50 centers across the US. At the time of writing this paper, the results of that trial have not been published; however, this trial was terminated prematurely because patients in the angioplasty/stent group fared worse than those in the medical therapy group. What has been released about this trial thus far implies that optimizing medical management remains the first treatment of choice for symptomatic intracranial atherosclerotic disease. The SAMMPRIS trial did not address those patients who had failed optimal medical therapy. Endovascular treatment in these patients may still play an important role in optimizing clinical outcome.

## 4. Discussion

ICAD remains a common cause of stroke worldwide, and our understanding regarding the optimal treatment of it remains dismal. The risk of ICAD-associated stroke increases with high-grade (>70%) stenoses and in those patients who demonstrate an increased OEF [25]. The conclusion that these patients need to be treated is likely a sound one, but the optimal therapy for this group desperately needs clarification.

 Aspirin, as the antithrombotic drug of choice, reduces the rate of recurrent ipsilateral stroke but does not eliminate it, and approximately 1 in 7 such patients will go on to experience recurrent ischemic events [4]. Patients with high-grade stenosis who also have an elevated OEF who experience recurrent strokes despite aspirin therapy are in need of revascularization procedures. The premature termination of the COSS study implies that microsurgical EC-IC bypass may not be the revascularization treatment of choice for these patients.

 Surgery, in the form of EC-IC bypass procedures is yet to be proven to lower recurrent strokes compared to medical management only. Previous trials may have been flawed by indiscriminate inclusion of subjects without the use of hemodynamic testing to determine who would more likely benefit from the procedure. The COSS and JET trials were initiated to investigate if indeed patients with an elevated OEF were more likely to benefit from surgery. While the final results of the JET trial are pending, the premature termination of the COSS trial implies the limited role of EC-IC bypass in this patient population.

Previous stents were largely designed for coronary disease, and the tortuous anatomy of the intracranial vasculature limited their utility. The advent of the Wingspan stent with its flexibility is quite promising, but the initial results of the SAMMPRIS trial comparing the use of the Wingspan stent with medical management imply that medical management remains the treatment of choice for patients with symptomatic ICAD. The role of angioplasty alone or angioplasty/stenting in the patient population that has failed optimal medical management remains to be determined. As of now, these patients have no other reliable alternative other than endovascular therapy.

## 5. Conclusion

Symptomatic ICAD is a significant health burden, and often times, leads to recurrent, disabling strokes. The natural history of ICAD is poorly understood, as are optimal treatment strategies. The results of long-term outcome studies assessing the utility of both microsurgical and endovascular treatment options continue to support the fact that optimizing medical management remains the initial treatment of choice. As refinements in both microsurgical and endovascular techniques/technologies continue, patients suffering from this devastating disease may have other alternatives.
